# Antibody–drug conjugates beyond HER2: Translating evidence into practice for HR+ HER2-negative and triple-negative metastatic breast cancer

**DOI:** 10.1016/j.breast.2026.104853

**Published:** 2026-06-30

**Authors:** Mariana Carvalho Gouveia, Pedro José Galvão Freire, Caio Guilherme Souza Prado, Caio Abner Leite, Bruna Migliavacca Zucchetti, Renata Colombo Bonadio, Carlos Barrios, Adriana Kahn, Maryam Lustberg, Romualdo Barroso-Sousa

**Affiliations:** a Yale Cancer Center, Yale School of Medicine, New Haven, United States, 333 Cedar Street, New Haven, CT, 06510, United States; b Clinical Oncology, Rede D'Or, Recife, Brazil, Avenida Agamenon Magalhães, 4760, Ilha do Leite, Recife, PE, 50070-160, Brazil; c Clinical Oncology, Cancer Institute of the State of São Paulo- ICESP, University of São Paulo- USP, São Paulo, Brazil, Avenida Dr. Arnaldo, 251, Cerqueira César, São Paulo, SP, 01246-000, Brazil; d Clinical Oncology, Brasília Hospital - Américas Oncologia, Brasília, Brazil, St. de Habitações Individuais Sul QI 15, Lago Sul, Brasília, DF, 70390-700, Brazil; e Clinical Oncology, Hospital 9 de Julho- Américas Oncologia, São Paulo, Brazil, Rua Peixoto Gomide, 545, Bela Vista, São Paulo, SP, 01309-001, Brazil; f Clinical Oncology, Instituto D'Or de Pesquisa e Ensino (IDOR), São Paulo, Brazil, Avenida República do Líbano, 2267, Ibirapuera, São Paulo, SP, 04501-000, Brazil; g Latin American Cooperative Oncology Group, Porto Alegre, Brazil, Rua Mostardeiro, 777, Moinhos de Vento, Porto Alegre, RS, 90430-001, Brazil

**Keywords:** Antibody–drug conjugates, Breast cancer

## Abstract

Antibody–drug conjugates (ADCs) have redefined therapeutic paradigms in metastatic breast cancer, offering unprecedented efficacy across hormone receptor–positive/HER2-negative and triple-negative subtypes. Trastuzumab deruxtecan, sacituzumab govitecan, datopotamab deruxtecan, and sacituzumab tirumotecan are expanding treatment options beyond traditional chemotherapy. As their clinical integration advances, optimizing the prevention and management of ADC-related toxicities, determining optimal sequencing strategies, and refining biomarker characterization have become central challenges. Ongoing research into ADC combinations, earlier-line use, and precision biomarker selection is expected to further reshape the therapeutic landscape and personalize care for patients with advanced breast cancer.

## Highlights

• ADCs have transformed the treatment of HER2-negative metastatic breast cancer.
• Key ADCs improve outcomes vs chemotherapy in selected settings and lines.
• Subtype, HER2 expression, patient/tumor features, toxicities may guide ADC choice.
• Optimal ADC sequencing remains an open and clinically relevant question.

## Graphical abstract

**Figure:**
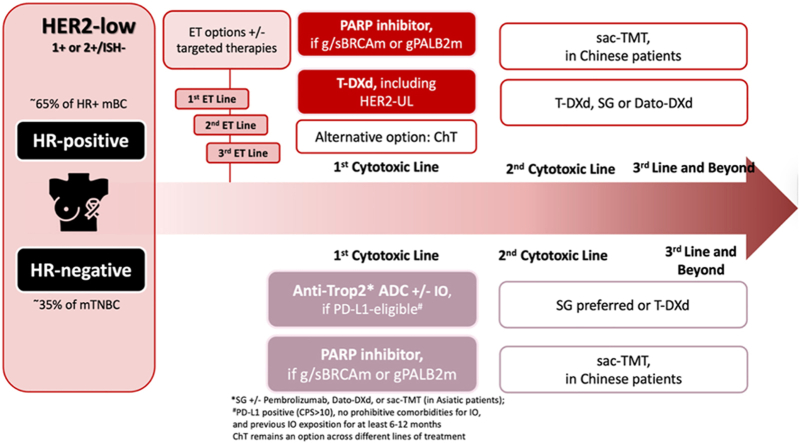

## 1 Introduction

Breast cancer is the most prevalent malignancy and leading cause of cancer-related mortality among women globally, encompassing distinct molecular subtypes with varied prognoses [1,2]. Among these, hormone receptor (HR)-positive, human epidermal growth factor receptor 2 (HER2)-negative breast cancer in its endocrine-refractory setting and triple-negative breast cancer (TNBC) pose significant therapeutic challenges in advanced disease, as both remain incurable with unfavorable outcomes [3]. Following progression on cyclin-dependent kinase 4/6 (CDK4/6) inhibitors, patients with HR-positive, HER2-negative metastatic breast cancer (mBC) derive limited benefit from subsequent treatments, with real-world median progression-free survival (PFS) below twelve months [4]. Similarly, even after incorporation of immunotherapy and PARP inhibitors, real-world median overall survival (OS) for metastatic TNBC remains approximately 12 months [5,6].

Antibody–drug conjugates (ADCs) have emerged as a disruptive therapeutic approach in breast cancer. These complex agents integrate a monoclonal antibody directed against a tumor-associated antigen, a cytotoxic payload, and a specialized linker with its own pharmacological properties [[7], [8], [9], [10]]. This design combines antibody specificity with potent cytotoxic delivery, broadening the therapeutic window. Advances in linker technology have overcome early limitations of first-generation ADCs, which were hindered by unstable linkers that prematurely released cytotoxic payloads into circulation, reducing efficacy and increasing systemic toxicity [11,12]. Despite these improvements, ADC-related toxicities persist and may be on-target—when the antigen is expressed in healthy tissues—or off-target, resulting from residual premature payload release [13,14]. Notably, cleavable linkers engineered to be processed by tumor-associated cathepsins enable effective extracellular payload release, enhancing antitumor activity even in cells lacking target antigen expression [15]. Additionally, the membrane-permeable nature of certain payloads allows diffusion into neighboring antigen-negative tumor cells—a phenomenon known as the bystander effect—which can further improve efficacy [13,14].

This narrative review provides a comprehensive overview of ADCs in metastatic HR-positive, HER2-negative and TNBC, focusing on translating clinical trial evidence into practice, including mechanisms of action, key trials, treatment-related toxicities, biomarkers, sequencing strategies and future perspectives..

**Fig. 1:**
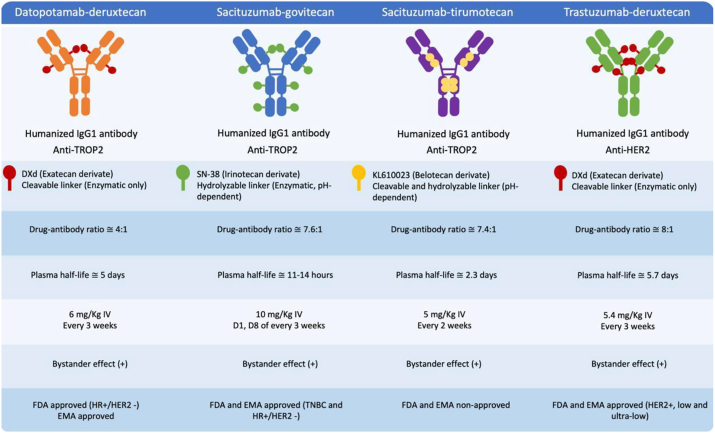
**Approved and late-phase ADCs in HR-positive, HER2-negative and triple-negative breast cancerAbbreviations:** DXd: Deruxtecan, FDA: Food and Drug Administration, EMA: European Medicines Agency. ∗Sacituzumab Tirumotecan is approved by National Medical Products Administration (NMPA) in China for TNBC.

## 2 Currently approved antibody drug-conjugates (Fig. 1)

### 2.1 Trastuzumab deruxtecan (T-DXd)

Trastuzumab deruxtecan (T-DXd, DS-8201) is a HER2-targeted ADC comprising a humanized anti-HER2 IgG1 monoclonal antibody with the same amino acid sequence as trastuzumab, conjugated via a cleavable tetrapeptide-based linker to a topoisomerase I inhibitor payload (a derivative of exatecan) with potency 10 times greater than SN-38 [[16], [17], [18]]. With a high drug-to-antibody ratio (DAR) of approximately 8, T-DXd is internalized upon HER2 binding, undergoes intracellular cleavage, and releases deruxtecan, inducing DNA damage and apoptosis. Preclinical data also suggest that extracellular linker cleavage by tumor-associated cathepsin L may enable payload release independently of HER2 binding and internalization, and T-DXd may promote immunogenic cell death with activation of innate immune signaling [15]. The payload's short half-life minimizes systemic exposure, while its high membrane permeability and lipophilic nature enable a potent bystander effect—destroying neighboring HER2-negative tumor cells—validated both in vitro and in vivo [[16], [17], [18]].

### 2.2 Sacituzumab govitecan (SG)

Sacituzumab govitecan (SG, IMMU-132) is an ADC targeting trophoblast cell-surface marker 2 (TROP2), a transmembrane protein overexpressed in many epithelial cancers including breast tumors. It conjugates an anti-TROP2 humanized antibody to SN-38, a topoisomerase I inhibitor, via a hydrolysable CL2A linker with intermediate serum stability, enabling both intracellular and extracellular payload release and contributing to a bystander effect. With a DAR of approximately 7.6, SG delivers high drug concentrations per antibody molecule. The intermediate linker stability results in a relatively short systemic half-life, necessitating administration on days 1 and 8 of a 21-day cycle to maintain therapeutic exposure [19,20].

### 2.3 Datopotamab deruxtecan (Dato-DXd)

Datopotamab deruxtecan (Dato-DXd, DS-1062a) is a TROP2-directed ADC comprising a humanized anti-TROP2 IgG1 monoclonal antibody linked to the same topoisomerase I inhibitor used in T-DXd via a tetrapeptide-based cleavable linker. Upon TROP2 binding and internalization, lysosomal processing releases the cytotoxic payload intracellularly. With a DAR of 4 and high plasma stability, Dato-DXd has a prolonged half-life allowing every-three-week dosing [[21], [22], [23]].

### 2.4 Sacituzumab tirumotecan (sac-TMT)

Sacituzumab tirumotecan (sac-TMT, SKB264/MK-2870) is an anti-TROP2 ADC sharing the same antibody backbone as SG but delivering a distinct payload—KL610023, a belotecan-derived topoisomerase I inhibitor—via a hydrolytically cleavable sulfonyl pyrimidine linker with improved circulatory stability. With a high DAR (∼7.4), sac-TMT enables dual extracellular and intracellular drug release. Upon TROP2 binding and receptor-mediated endocytosis, lysosomal cleavage releases KL610023, inducing DNA double-strand breaks, cell cycle arrest, and apoptosis. The membrane-permeable payload further diffuses into the tumor microenvironment, exerting bystander cytotoxicity on neighboring cells with low or heterogeneous TROP2 expression [24,25]..

**Table 1:** Summary of adverse events of the currently approved ADCs evaluated in hormone receptor-positive, HER2-negative and triple-negative breast cancer.

Drug (Trial)	T-DXd (Destiny-Breast04) [26] **^N^^= 371^**		T-DXd (Destiny-Breast06) [27] **^N^^= 436^**		Dato-DXd (TROPION-Breast 01) [28] **^N^^= 365^**		Dato-DXd (TROPION-Breast 02) [29] **^N^^= 323^**		SG (TROPiCS-02) [30] **^N^^= 272^**		SG (ASCENT) [31] **^N^^= 235^**		SG (EVER) [32] **^N^^= 166^**		SG (ASCENT- 03) [33] **^N^^= 279^**		SG + Pembro (ASCENT-04) [34] **^N^^= 221^**		Sac-TMT (OptiTROP Breast01) [35] **^N^^= 130^**		Sac-TMT (OptiTROP Breast02) [36] **^N^^= 200^**	
Adverse event (%)	Any	≥G3	Any	≥G3	Any	≥G3	Any	≥G3	Any	≥G3	Any	≥G3	Any	≥G3	Any	≥G3	Any	≥G3	Any	≥G3	Any	≥G3
Neutropenia	33	14	38	21	11	1	12	3	70	51	63	51	88	69	67	43	63	43	79	35	80	45
Anemia	33	8	28	6	11	1	15	2	34	6	35	8	71	18	39	4	37	7	82	29	84	13
Thrombocytopenia	24	5	-	-	-	-	-	-	6	1	5	2	20	4	4	1	5	1	41	13	38	7
Nausea	73	5	66	2	52	1	45	<1	55	1	57	3	58	2	61	2	68	3	40	0	39	0
Vomiting	34	1	27	1	20	1	20	1	19	1	29	1	36	2	25	2	29	1	35	0	26	0
Diarrhea	22	1	24	2	-	-	-	-	57	9	59	11	51	7	54	9	70	10	-	-	-	-
Fatigue	48	8	47	4	24	2	32	3	38	6	45	3	35	7	47	3	58	8	-	-	-	-
Alopecia	38	0	45	0	36	0	41	0	46	0	-	-	62	0	55	0	52	0	-	-	30	0
Stomatitis	-	-	-	-	50	6	57	8	-	-	-	-	-	-	-	-	-	-	49	10	63	10
Dry eye	-	-	-	-	22	1	24	1	-	-	-	-	-	-	-	-	-	-	-	-	-	-
Pneumonitis	12	2	11	1	3	1	-	-	0	0	1	1	-	-	-	-	-	-	-	-	-	-

**Abbreviations:** ADC: Antibody–drug conjugates, T-DXd: Trastuzumab Deruxtecan, Dato-DXd: Datopotamab Deruxtecan, SG: Sacituzumab govitecan, Pembro: Pembrolizumab, Sac-TMT: Sacituzumab Tirumotecan, Any: All grades, G: Grade, -: Not reported.

**Table 2:** Summary of prophylactic measures for the management of ADC toxicities.

Adverse Event	General Prophylaxis	Management (All ADCs)	ADC Most Associated
**Neutropenia**	Primary G-CSF prophylaxis recommended for SG in high-risk patients or for recurrent cases	Hold for ≥ G3 until recovery to ≤ G2; resume with dose reduction if recurrent;	SG – highest rates of neutropenia
**Nausea/Vomiting**	5-HT3 antagonist + dexamethasone + NK1 antagonist ± olanzapine	Standard antiemetic prophylaxis; high emetogenic risk regimens require 3–4 drug prophylaxis	T-DXd – highest rates of nausea/vomiting
**Mucositis**	Oral hygiene, corticosteroid mouthwashes (established for Dato-DXd; extrapolated for Sac-TMT)	Hold for ≥ G3; resume with dose reduction; symptomatic treatment if mild	Dato-DXd and Sac-TMT – highest rates of mucositis
**Diarrhea**	Loperamide; atropine if early onset (for SG)	Symptomatic management; hold for ≥ G3; resume with dose reduction if recurrent	SG – highest rates of diarrhea
**Pneumonitis/ILD**	Monitor symptoms, consider early CT chest if suspicion	Hold at first symptoms; discontinue for ≥ G2; initiate corticosteroids (prednisone 0.5–1 mg/kg/day)	T-DXd – highest rates of ILD/pneumonitis; CT chest should be performed every 6-12 weeks

**Abbreviations:** ADC – Antibody-drug conjugate, T-DXd – Trastuzumab deruxtecan, SG – Sacituzumab govitecan, Dato-DXd – Datopotamab deruxtecan, Sac-TMT – Sacituzumab tirumotecan, G-CSF – Granulocyte-colony stimulating factor, ILD – Interstitial lung disease, CT – Computed tomography.

## 3 Toxicity profile and practical considerations for prevention and/or management of adverse events associated with different ADCs (Table 1, Table 2)

### 3.1 Trastuzumab deruxtecan

T-DXd is associated with clinically significant toxicities. The most common adverse events include nausea, vomiting, fatigue, and alopecia, while grade ≥3 toxicities occur in approximately 52% of patients, predominantly neutropenia, anemia, and fatigue. Hematologic toxicities require close monitoring and may necessitate dose modifications or growth factor support [37,38]. Asthenia or fatigue, often cumulative, should be addressed through supportive care including non-pharmacological interventions and dose adjustments [37,38].

Given its high emetogenic potential, T-DXd is classified by the NCCN as a high-risk agent for nausea and vomiting. Three Category 1 antiemetic strategies are recommended for highly emetogenic regimens: a preferred four-drug regimen consisting of olanzapine, an NK1 receptor antagonist, a 5-HT3 receptor antagonist, and dexamethasone; a three-drug regimen of olanzapine, palonosetron, and dexamethasone; or a three-drug regimen comprising an NK1 receptor antagonist, a 5-HT3 receptor antagonist, and dexamethasone. Prolonged nausea beyond the acute phase has been reported, underscoring the need for extended antiemetic coverage in selected patients [39,40].

A key safety concern is interstitial lung disease (ILD)/pneumonitis, reported in approximately 12% of patients, with fatal cases in 1%. ILD is the most frequent cause of permanent treatment discontinuation (∼8%), while overall discontinuation due to adverse events is approximately 15%. Early detection through patient education on respiratory symptoms and periodic chest imaging (every 6–12 weeks) is recommended. Per prescribing information, T-DXd may be restarted after complete resolution of grade 1 ILD. In a pooled analysis of 45 retreated patients with grade 1 ILD, recurrence occurred in 33.3%, but all events were grade 1–2 with no fatal cases, supporting the safety of rechallenge after grade 1 ILD [41]. Current guidelines recommend permanent discontinuation for grade ≥2 ILD [37,38]. Real-world data from 9 patients rechallenged after grade 2 ILD showed recurrence in 22%, with only 1 grade 3 event [42]. Emerging data have identified potential risk factors for ILD, including prior abemaciclib exposure, smoking, prior immunotherapy, and inherited genetic variation in CYP3A4/CYP3A5, which may inform future risk stratification strategies [[43], [44], [45], [46]].

### 3.2 Sacituzumab govitecan

The safety profile of SG is characterized primarily by hematologic and gastrointestinal toxicities, including neutropenia, diarrhea, nausea, fatigue, and alopecia. Grade ≥3 neutropenia and diarrhea occur in approximately 50% and 10% of patients, respectively; however, the treatment discontinuation rate remains low (∼5%), reflecting overall tolerability with appropriate supportive care [47].

Routine prophylaxis with G-CSF and loperamide was not initially recommended in pivotal trials; however, data from the phase II PRIMED study support their prophylactic use, demonstrating meaningful reductions in early-cycle neutropenia and diarrhea [47,48]. With extended follow-up, grade ≥3 neutropenia occurred in 24.0% and grade ≥2 diarrhea in 18.0% of patients receiving prophylaxis [49]. For acute diarrhea or early cholinergic syndrome during or shortly after infusion, intravenous atropine (0.4 mg every 15 min, up to 2 doses) is recommended [49]. The NCCN classifies SG as a high emetogenic risk agent, warranting antiemetic prophylaxis — either the preferred four-drug regimen or one of two triplet alternatives [39]. Clinical experience suggests that nausea may be less pronounced with SG compared to T-DXd.

SN-38 is metabolized via UGT1A1, and the UGT1A1∗28 homozygous polymorphism is associated with significantly increased risk of neutropenia, anemia, and grade ≥3 toxicities (OR 7.03), with higher rates of dose reductions and treatment interruptions. Despite this, pre-treatment UGT1A1 genotyping is not currently required [50].

### 3.3 Datopotamab deruxtecan

Dato-DXd demonstrates a favorable safety profile with a lower incidence of grade ≥3 adverse events compared to chemotherapy, and low treatment discontinuation rate (2.5%). The most common adverse events include nausea, stomatitis, alopecia, and fatigue, with stomatitis being the most frequent grade ≥3 toxicity (6.4%). Dato-DXd is also classified as a high emetogenic risk agent, warranting preventive antiemetic measures. As it tends to be less emetogenic than T-DXd and SG in clinical practice, an initial triplet regimen may be sufficient, though the four-drug regimen remains an option [39,51].

Proactive stomatitis management is a cornerstone of supportive care, including regular oral hygiene, steroid-based mouthwash (preferably alcohol-free), and patient education on symptom recognition. Dose modifications should be applied for grade ≥2 events per protocol guidance [51].

Ocular surface events, likely related to TROP2 expression in corneal and conjunctival epithelium, occur in approximately 25% of patients, predominantly as dry eye and keratitis. Most are low-grade and manageable, though grade 3 keratitis occurred in 1.1%. Prophylactic preservative-free lubricating eye drops and avoidance of contact lenses are recommended from treatment initiation, with ophthalmologic evaluations every three cycles. Drug-related ILD rates were low (3.3%), consistent with prior Dato-DXd data [51].

### 3.4 Sacituzumab tirumotecan

The safety profile of Sac-TMT is characterized by hematologic and gastrointestinal toxicities. Anemia, neutropenia, and thrombocytopenia are the predominant adverse events, managed with supportive measures including secondary G-CSF prophylaxis and dose modifications. Despite frequent cytopenias, treatment discontinuation rates have remained low [35,36].

Gastrointestinal toxicities—nausea, diarrhea, and stomatitis—are usually low-grade and manageable with oral care protocols and moderate-risk antiemetic prophylaxis. Notably, stomatitis is a relevant grade ≥3 adverse event, with rates exceeding those observed with other TROP2-directed ADCs [35,36]. Unlike Dato-DXd, for which expert consensus prophylaxis protocols have been established, no Sac-TMT-specific oral prophylaxis guidelines currently exist. Prophylactic measures, including oral hygiene optimization and steroid-containing mouthwash, are extrapolated from other ADC settings and emerging preliminary data; dedicated recommendations are expected as clinical experience broadens [51]. Peripheral neuropathy and ILD have been infrequent, further supporting overall tolerability [35].

Importantly, current safety data derive primarily from East Asian populations. Given pharmacogenomic differences and other factors that may alter drug metabolism and toxicity risk, these results may not fully extrapolate to Western populations, and future studies in diverse cohorts are needed [35,36].

## 4 Efficacy data in HR-positive, HER2-negative breast cancer

### 4.1 Trastuzumab deruxtecan

T-DXd was evaluated in HER2-low mBC in the DESTINY-Breast04 trial, which randomized 557 patients (2:1) who had received one or two prior chemotherapy lines to T-DXd or chemotherapy of physician's choice. The population comprised 88% HR-positive tumors, with a median of three prior lines for metastatic disease, including endocrine therapy; 64% had received prior CDK4/6 inhibitors. T-DXd significantly improved both PFS and OS. In the HR-positive cohort, median PFS was 10.1 versus 5.4 months (HR 0.51; 95% CI, 0.40–0.64; P < 0.001), and median OS was 23.9 versus 17.5 months (HR 0.64; 95% CI, 0.48-0.86 P = 0.003) [26].

DESTINY-Breast06 evaluated T-DXd in an earlier treatment setting, enrolling chemotherapy-naïve patients exclusively with HR-positive mBC who had progressed on prior endocrine-based therapy, with a median of two previous endocrine-based lines. Notably, the trial expanded eligibility beyond HER2-low to include patients with HER2-ultralow expression (IHC 0 with any membrane staining 10%), a population previously excluded from ADC therapy. Eligibility required progression after at least two prior endocrine lines, or one line if criteria for primary endocrine resistance were met. A total of 866 patients were randomized (1:1) to receive T-DXd or physician's choice of chemotherapy; 713 (82%) and 153 (18%) had, respectively, HER2-low and HER2-ultralow tumors. Patients had a median of two previous lines of endocrine-based therapy for metastatic disease, and the majority had received CDK4/6 inhibitors for metastatic disease (90%). Only 3% had bone-only disease. T-DXd significantly improved PFS in the HER2-low population (13.2 vs. 8.1 months; HR 0.62; 95% CI, 0.52–0.75; P < 0.001), with consistent results in the ITT population (HR 0.64; 95% CI, 0.54–0.76; P < 0.001). In the exploratory HER2-ultralow subgroup, median PFS was numerically similar (13.2 vs. 8.3 months; HR 0.78; 95% CI, 0.50–1.21), suggesting T-DXd activity extends to tumors with minimal HER2 expression [27].

Results consistently favored T-DXd across HER2 expression levels (HER2-low, intention-to-treat and ultralow), with ORRs of 56.5%, 57.3%, and 61.8%, respectively. These findings supported the expanded approval of T-DXd for HR-positive, HER2-low or ultralow mBC in endocrine-resistant, chemotherapy-naïve patients [27]. Although OS data remain immature, exploratory subgroup analyses showed consistent efficacy, with particularly notable benefit in patients with higher risk, such as rapid progression (6 months) on prior CDK4/6 inhibitors (median PFS 14.0 vs. 6.5 months; HR 0.38; 95% CI, 0.25–0.59; ORR 67.7% vs. 25.4%) [52].

### 4.2 Sacituzumab govitecan

SG was evaluated in heavily pretreated HR-positive, HER2-negative mBC in the phase 3 TROPiCS-02 trial. Among 543 patients randomized (1:1) to SG or physician's choice chemotherapy—all previously exposed to CDK4/6 inhibitors and with a median of three prior chemotherapy lines—SG significantly improved PFS (5.5 vs. 4.0 months; HR 0.66; 95% CI, 0.53–0.83; P = 0.0003) and OS (14.4 vs. 11.2 months; HR 0.79; 95% CI, 0.65–0.96; P = 0.020), with a higher ORR (21% vs. 14%) [30,53]. Exploratory analyses showed benefit regardless of TROP2 expression (H-score 100: HR 0.77; ≥100: HR 0.60) and across HER2 expression levels, including HER2-low (PFS HR 0.58) and HER2-null (PFS HR 0.72) subgroups [54,55].

The phase 3 EVER-132-002 trial validated SG in a pretreated Asian population with a median of three prior chemotherapy lines, where only half had received prior CDK4/6 inhibitors, reflecting the evolving treatment landscape in Asia. Although statistically significant, the PFS difference was not clinically relevant (4.3 vs. 4.2 months; HR 0.67; P = 0.0028); however, SG demonstrated a meaningful OS benefit (21.0 vs. 15.3 months; HR 0.64; P = 0.0061) [32].

SG was also evaluated in a US-based Western population in combination with pembrolizumab in the phase II SACI-IO trial that enrolled patients with HR-positive, HER2-negative mBC previously treated with at least one line of endocrine therapy and up to one line of chemotherapy in the metastatic setting. A total of 104 patients were randomized to receive SG plus pembrolizumab or SG monotherapy. The majority had received prior CDK4/6 inhibitors (76.9%), and more than half (55.8%) had not received chemotherapy in the metastatic setting. The study demonstrated no significant difference in median progression-free survival (8.4 months with the combination vs. 6.2 months with SG alone; HR 0.76, 95% CI 0.47–1.23; p = 0.26). Objective response rates were 21.2% and 17.3%, respectively, and overall survival data remain immature with a median of 16.9 months for the combination and 17.1 months for SG alone [56].

Considering its efficacy in later-line settings, SG was subsequently evaluated in the chemotherapy-naïve setting in the phase 3 ASCENT-07 trial, which randomized 690 endocrine-refractory patients (2:1) to SG or single-agent chemotherapy. The population had high-risk features: a median of two prior endocrine therapy lines, 91% prior CDK4/6 inhibitor exposure (42% for 12 months), 88% visceral disease, and only 4% bone-only disease. The trial did not meet its primary endpoint by BICR (median PFS 8.3 months in both arms; HR 0.85; 95% CI, 0.69–1.05; P = 0.130). Early censoring within the first four months (8% in the SG arm, 11% in the chemotherapy arm) and high crossover to ADCs in the control group (43% vs. 16%) may have confounded results. Investigator-assessed PFS favored SG (8.4 vs. 6.4 months; HR 0.78; 95% CI, 0.64–0.93; P = 0.008), and an early OS trend was observed (HR 0.72; 95% CI, 0.54–0.97; P = 0.029), though OS data remain immature (27% maturity) [57]. While ASCENT-07 did not establish SG superiority in the pre-chemotherapy setting, SG remains a standard-of-care option after prior endocrine therapy and chemotherapy based on TROPiCS-02 [30].

### 4.3 Datopotamab deruxtecan

Dato-DXd was evaluated in the phase 3 Tropion-Breast01 trial, which randomized 732 patients with HR-positive, HER2-negative mBC—progressive after endocrine therapy and one or two prior chemotherapy lines—to Dato-DXd or investigator's choice chemotherapy. Most patients had received prior CDK4/6 inhibitors (82%), and 62% had one prior chemotherapy line. The trial met its co-primary endpoint of PFS (6.9 vs. 4.9 months; HR 0.63; 95% CI, 0.52–0.76; P 0.001), with improved ORR (36.4% vs. 22.9%). However, OS was not significantly different after a median follow-up of 22.8 months (18.6 vs. 18.3 months; HR 1.01; P = 0.94), potentially confounded by unplanned subsequent ADC use in the chemotherapy arm (24% vs. 12%). An inverse-probability of censoring weighting (IPCW) sensitivity analysis adjusting for subsequent ADC therapy showed a non-significant trend favoring Dato-DXd (HR 0.86; 95% CI, 0.70–1.06) [58].

### 4.4 Sacituzumab tirumotecan

In the phase 3 OptiTROP-Breast02 trial, 399 patients with HR-positive, HER2-negative mBC who had received 1–4 prior chemotherapy lines, prior taxane exposure, and at least one endocrine therapy plus CDK4/6 inhibitor were randomized (1:1) to sac-TMT or investigator's choice chemotherapy. Among enrolled patients, 47% had HER2-low disease, 16% had received ≥3 prior chemotherapy lines, 67% had received CDK4/6 inhibitors for less than one year, and 26% met criteria for endocrine resistance. At a prespecified interim analysis (median follow-up 7.4 months), sac-TMT significantly improved PFS (8.3 vs. 4.1 months; HR 0.35; 95% CI, 0.26–0.48; P 0.0001) and ORR (41.5% vs. 24.1%), with consistent benefit across subgroups. Notably, this interim PFS analysis included only patients with at least one post-baseline tumor assessment. An OS trend favoring sac-TMT was observed, though data remain immature [36].

## 5 Efficacy data in triple-negative breast cancer (TNBC)

### 5.1 Sacituzumab govitecan

The pivotal phase 3 ASCENT trial randomized 529 patients with relapsed/refractory metastatic TNBC (≥2 prior lines including a taxane) to SG or physician's choice chemotherapy. Approximately 30% had received more than 3 prior lines of therapy, and previous treatment included immunotherapy in 27% of cases. SG significantly improved PFS (5.6 vs. 1.7 months; HR 0.41; 95% CI, 0.32–0.52; P0.001) and OS (12.1 vs. 6.7 months; HR 0.48; 95% CI, 0.38–0.59; P0.001), with consistent benefit across TROP2 expression levels. PD-L1 expression data were not reported [31].

Building on these results and the established role of first-line chemo-immunotherapy for PD-L1–positive TNBC (KEYNOTE-355) [59], the phase 3 ASCENT-04 trial randomized 443 patients with PD-L1–positive metastatic TNBC (1:1) to SG plus pembrolizumab or chemotherapy plus pembrolizumab. Among patients, 34% had de novo metastatic disease and 48% recurred >12 months after taxane-based curative therapy. SG plus pembrolizumab significantly improved PFS (11.2 vs. 7.8 months; HR 0.65; 95% CI, 0.51–0.84; P = 0.0009), ORR (60% vs. 53%), and median duration of response (16.5 vs. 9.2 months). As OS data remain immature, and interpretation will be influenced by crossover and attrition bias inherent to first-line TNBC trials, PFS2 provides an important measure of long-term clinical benefit. Crossover was not only permitted but actively facilitated: the sponsor provided SG to participating study sites, ensuring access even in settings where SG would otherwise not have been commercially available. Among the 170 patients in the chemotherapy plus pembrolizumab arm who discontinued treatment, 119 received subsequent therapy, most commonly SG (81%). Despite this high crossover rate, PFS2 remained significantly longer in the SG plus pembrolizumab arm, with a 33% reduction in the risk of a PFS2 event compared with chemotherapy plus pembrolizumab (HR, 0.67; 95% CI, 0.48–0.95). Median PFS2 was not reached in the SG plus pembrolizumab arm versus 21.0 months in the chemotherapy plus pembrolizumab arm [60]. Patient-reported outcomes also favored the SG combination for physical, role, and emotional functioning [34,61].

For patients ineligible for immunotherapy, the phase 3 ASCENT-03 trial randomized 558 patients (99% PD-L1–negative) to first-line SG or standard chemotherapy. Among enrolled patients, 31% had de novo metastatic disease, 5% had brain metastases, 20% recurred within 6–12 months after curative-intent treatment, and 48% recurred more than 12 months after curative therapy. SG significantly improved PFS (9.7 vs. 6.9 months; HR 0.62; 95% CI, 0.50–0.77; P0.001), with comparable ORR between arms (48% vs. 46%) [33]. Similarly to ASCENT-04, crossover to SG was permitted and sponsor-facilitated. Of the 240 patients who discontinued chemotherapy, 179 received subsequent treatment, most frequently SG (82%). Despite this, median PFS2 was significantly prolonged with first-line SG compared with chemotherapy (18.2 vs. 14.0 months; HR, 0.70; 95% CI, 0.55–0.90), reinforcing SG as a new standard in this setting; mature OS data are awaited [62].

### 5.2 Sacituzumab tirumotecan

The phase 3 OptiTROP-Breast01 trial, conducted across 49 sites in China, randomized 263 patients with relapsed/refractory metastatic TNBC (≥2 prior systemic lines) to sac-TMT or physician's choice chemotherapy. Most had visceral metastases (86.7%), and nearly half (47.5%) had received ≥3 prior chemotherapy regimens. Sac-TMT significantly improved PFS (6.7 vs. 2.5 months; HR 0.32; 95% CI0.24-0.44; P < 0.00001), OS (not reached vs. 9.4 months; HR 0.53; P = 0.0005), and ORR (45.4% vs. 12.0%). Benefit was consistent across subgroups including low TROP2 expression and prior immunotherapy exposure. These results led to the approval of sac-TMT in China [35].

At ASCO 2025, the phase 2 OptiTROP-Breast05 trial evaluated sac-TMT as first-line therapy in 41 patients with advanced TNBC irrespective of PD-L1 status (78% PD-L1 negative). The ORR was 70.7% with a median PFS of 13.4 months; in the PD-L1–negative subgroup, ORR was 71.9% and median PFS 13.1 months, supporting sac-TMT activity in the first-line setting regardless of PD-L1 expression [63].

### 5.3 Datopotamab deruxtecan

Early evidence for Dato-DXd in TNBC emerged from the phase 1 TROPION-PanTumor01 study, which demonstrated encouraging antitumor activity even in heavily pretreated TNBC, with an ORR of 31.8% and median PFS of 4.4 months among 44 patients (7.3 months in the topoisomerase I–naive subgroup) [64]. These findings prompted further evaluation in the phase 1b/2 BEGONIA trial, where Dato-DXd plus durvalumab achieved an ORR of 79.0% in Arm 7 (n = 62; 87% PD-L1–negative) with a median PFS of 14.0 months, and 81.8% in Arm 8 (n = 33; PD-L1–high). Notably, the high response rates observed despite the predominantly PD-L1–negative population challenged the assumption that immunotherapy benefit requires PD-L1 expression [65].

The phase 3 TROPION-Breast02 trial evaluated Dato-DXd as monotherapy in the first-line setting for 644 patients with metastatic TNBC ineligible for immunotherapy, randomized to Dato-DXd or investigator's choice of single-agent chemotherapy (paclitaxel, nab-paclitaxel, eribulin, capecitabine, or carboplatin). Among enrolled patients, 90% were PD-L1–negative, 10% had brain metastases, 34% had de novo metastatic disease, and among those with recurrent disease, 15%, 6%, and 45% had a disease-free interval of 6, 6–12, and >12 months, respectively. Dato-DXd met both co-primary endpoints, significantly improving PFS (10.8 vs. 5.6 months; HR 0.57; 95% CI, 0.44–0.73; P < 0.0001) and OS (23.7 vs. 18.7 months; HR 0.79; 95% CI, 0.64–0.98; P = 0.029) compared with chemotherapy, with no crossover permitted. The confirmed ORR was 62.5% versus 29.3% [29].

Secondary efficacy endpoints were recently presented and published.47,48 PFS2 was significantly improved with Dato-DXd (median 15.6 vs. 11.8 months; HR 0.61; 95% CI, 0.50–0.74). Approximately 75% of patients received subsequent therapy, with ADCs used more frequently in the chemotherapy arm (41% vs. 21%). Time to first subsequent therapy or death (TFST: median 10.9 vs. 5.6 months; HR 0.49; 95% CI, 0.41–0.59) and time to second subsequent therapy or death (TSST: median 16.7 vs. 12.6 months; HR 0.67; 95% CI, 0.55–0.81) were both improved with Dato-DXd, consistent with the primary endpoints and further supporting a sustained first-line benefit despite crossover to ADCs in the control arm [66,67].

### 5.4 Trastuzumab deruxtecan

Early-phase data first suggested antitumor activity of T-DXd in heavily pretreated HER2-low breast cancer, though response rates were notably lower in TNBC compared to HR-positive disease [68].

The DESTINY-Breast04 as mentioned above, evaluated HER2–negative mBC with low expression of HER2, defined as a score of 1+ on IHC analysis or as an IHC score of 2+ and negative ISH, including HR–positive and HR–negative tumors that had received one or two previous lines of chemotherapy. However, among 557 patients included, only 63 were TNBC patients. The median PFS in this cohort was 8.5 months in the T-DXd arm and 2.9 months in the chemotherapy group (HR 0.46; 95% CI, 0.24 -0.89). Within this subgroup, the median OS was 18.2 months (95% CI, 13.6 to not evaluable) with T-DXd group and 8.3 months in the physician's choice arm (HR 0.48; 95% CI, 0.24-0.95) [26]. Considering these results, T-DXd is also an option for patients with HR-negative HER2-low tumors..

**Fig. 2:**
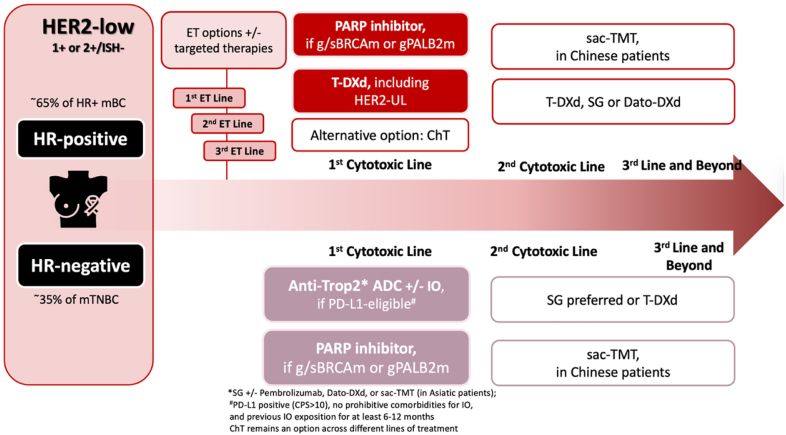
Suggested algorithm in the treatment of HER2-low triple-negative breast cancer and HR-positive, HER2-negative breast cancer # This is a suggested algorithm that does not consider regulatory approval. Abbreviations: ADC: Antibody-drug conjugate, HR: hormone receptor, HER2: human epidermal growth factor receptor 2, mBC: metastatic breast cancer, mTNBC: metastatic triple-negative breast cancer, ET: endocrine therapy, T-DXd: Trastuzumab Deruxtecan, HER2-UL: HER2-ultralow; IO: immunotherapy, SG: Sacituzumab Govitecan, Dato-DXd: Datopotamab Deruxtecan, sac-TMT: Sacituzumab tirumotecan, g/sBRCAm: germline or somatic BRCA1/2 mutation, gPALB2: germline PALB2 mutation, ChT: chemotherapy.

## 6 How to select the agents in clinical practice? (Fig. 2)

The following treatment considerations are based on the available clinical evidence and current expert opinion. In the absence of head-to-head randomized trials comparing ADCs, several recommendations rely on indirect cross-trial comparisons and should be interpreted with caution.

### 6.1 HR-positive, HER2-negative breast cancer

Treatment sequencing in HR-positive, HER2-negative mBC beyond CDK4/6 inhibitor progression is rapidly evolving and depends on several clinical and molecular factors, including time on first-line endocrine-based therapy, symptom burden, disease extent, germline BRCA1/2 status, tumor mutations (PIK3CA, AKT, PTEN, ESR1), and HER2 expression (low or ultralow by immunohistochemistry).

For patients with endocrine-sensitive disease and prolonged benefit (preferably >12 months) on first-line CDK4/6 inhibitor plus endocrine therapy, particularly those harboring somatic PIK3CA, AKT, PTEN, or ESR1 mutations, a subsequent endocrine-based regimen is recommended. Conversely, for patients with endocrine-resistant or symptomatic disease after first-line CDK4/6 inhibitor therapy, ADCs have emerged as alternative treatment options.

#### 6.1.1 HER2-low or ultra-low disease

Based on data from pivotal ADC trials in HR-positive, HER2-negative mBC, T-DXd is the preferred option over anti-TROP2 ADCs, for patients with HER2-low tumors when ADC therapy is indicated, provided there are no contraindications such as pre-existing pneumopathy. In this subgroup, results of DESTINY-Breast04 and DESTINY-Breast06 demonstrated a consistent efficacy, with significant improvement in both PFS and OS in second line as well as a PFS improvement in chemotherapy naïve patients [26,27]. Despite these results, the optimal timing for T-DXd use remains uncertain. In clinical practice, T-DXd is strongly favored after endocrine therapy for patients with symptomatic disease, rapid progression (within six months of CDK4/6 inhibitor therapy), or high tumor burden—clinical scenarios where delaying an effective treatment could compromise performance status and result in missed opportunities for ADC in later lines. For patients with HER2-ultralow disease, DESTINY-Breast06 included a small cohort, but demonstrated a PFS exploratory benefit comparable to that seen in HER2-low tumors, suggesting that this subgroup also derives benefit. Since patients with HER2-ultralow expression were excluded from DESTINY-Breast04, T-DXd is not currently supported for later-line use in this population, making its use in the chemotherapy-naïve setting the most appropriate option. In parallel, strategies involving both emerging and established ADCs are also being explored in the first-line setting (Table 3A).

**Table 3:** Phase 3 first-line ADC trials in breast cancer.

**3A. Triple-negative breast cancer (1L mTNBC)**					
**Trial (NCT)**	**PD-L1 stratum**	**ADC/Arms**	**N (planned/enrolled)**	**Primary endpoint/Status**	**Results**
ASCENT-03 (NCT05382299) [33]	PD-L1 negative or ICI-ineligible	SG vs TPC chemo	558	PFS	Positive for PFS 8.1 vs 5.6 m HR 0.62 (CI 0.50-0.77) P < 0.001
ASCENT-04/KEYNOTE-D19 (NCT05382286) [34]	PD-L1 positive (CPS ≥10; 22C3)	SG + pembro vs chemo + pembro	443	PFS	Positive for PFS 11.2 vs 7.8 m HR 0.65 (CI 0.51-0.84) P < 0.0009)
TROPION-Breast02 (NCT05374512) [29]	PD-L1 negative or ICI-ineligible	Dato-DXd vs TPC chemo	644	PFS OS	Positive for PFS 10.8 vs 5.6 m HR 0.57 (CI 0.47-0.69) P < 0.0001 Positive for OS 23.6 VS 18.7 m HR 0.79 (CI 0.64-0.98) P 0.0291
TROPION-Breast05 (NCT06103864)	PD-L1 positive (CPS ≥10; 22C3)	Dato-DXd ± durvalumab vs chemo + pembro	∼675	PFS (Ongoing)	Not yet Published
TroFuse-011 (NCT06841354)	All-comers	Sac-TMT ± pembro vs TPC chemo	∼1000	PFS (Ongoing)	Not yet Published

**3B. HR-positive/HER2-negative (1L setting after endocrine therapy)**				
Trial (NCT)	ADC/Arms	N (planned/enrolled)	Primary endpoint/Status	**Results**
DESTINY-Breast06 (NCT04494425) [27]	T-DXd vs TPC chemo	866 total (794 HR+/HER2-low/ultralow)	PFS	Positive for PFS 13.2 vs 8.1 m HR 0.62 (CI 0.52-0.75) P < 0.001)
ASCENT-07 (NCT05840211) [57]	SG vs TPC chemo (first-line chemo after ET resistance)	690 (central assessments: Trop2, HR and HER2)	PFS by BICR	Negative for PFS 8.3 vs 8.3 m HR 0.85 (0.69-1.05) P 0.130
TroFuse-010 (NCT06312176)	sac-TMT vs sac-TMT + Pembro vs TPC Chemo	~1200 planned (central assessments: Trop2, PD-L1, HR and HER2)	PFS (Ongoing)	Not yet Published
HERTHENA-Breast04 (NCT07060807)	Patri-DXd vs TPC chemo (or T-DXd)	~1000 planned (central assessments: HER3, HR and HER2)	PFS and OS (Ongoing)	Not yet Published

**Abbreviations:** ADC: Antibody-drug conjugate; SG: Sacituzumab govitecan; Dato-DXd: Datopotamab deruxtecan; T-DXd: Trastuzumab deruxtecan; Sac-TMT: Sacituzumab tirumotecan; TPC: Treatment of physician's choice; ICI: Immune checkpoint inhibitor; PFS: Progression-free survival; Patri-DXd: Patritumab-deruxtecan; OS: Overall survival; CPS: Combined positive score; Pembro: Pembrolizumab; BICR: Blinded Independent Central Review; m: months; HR: hazard ratio; vs: versus.

For patients with indolent disease or low tumor burden who have exhausted endocrine-based options or whose disease is considered endocrine-resistant, chemotherapy prior to ADC remains a therapeutic option. This is particularly relevant given that the DESTINY-Breast06 trial primarily enrolled patients with visceral disease, with only 3% of participants having bone-only metastases [27]. For these patients, several chemotherapy options, including capecitabine, paclitaxel, and nab-paclitaxel, may be considered. Capecitabine, as an oral agent, may be particularly attractive for selected patients because it avoids alopecia and is associated with a favorable toxicity profile. Nevertheless, T-DXd remains an appropriate alternative even in this setting, as subgroup analyses of DESTINY-Breast06, patients with no visceral disease had a progression-free survival of 23.3 months versus 11.3 months with chemotherapy (HR 0.51, 95% CI 0.30 – 0.85). This highlights the importance of tailoring therapeutic strategies to individual disease characteristics, ensuring that treatment aligns with both clinical needs and patient preferences. [52].

T-DXd is also being evaluated in combination strategies in the DESTINY-Breast08 study (NCT04556773), investigating its use with durvalumab plus paclitaxel, capivasertib, anastrozole, fulvestrant, and capecitabine in HER2-low mBC. Also, the phase II PONTIAC trial (NCT06486883) is evaluating T-DXd versus CDK4/6 inhibitor-based endocrine therapy as first-line treatment in HR-positive, HER2-low/ultralow advanced breast cancer patients classified as non-luminal by intrinsic subtyping, testing whether molecular subtype may identify patients who benefit from early ADC use over conventional endocrine-based strategies.

#### 6.1.2 HER2 0 without any membrane expression (HER2-null)

Patients with HER2-null and no detectable membrane staining were excluded from both DESTINY-Breast04 and DESTINY-Breast06, making T-DXd unsupported in this population [26,27]. For these patients, SG and Dato-DXd represent suitable alternatives after progression on endocrine therapy and at least one prior line of chemotherapy, as both demonstrated improved PFS and higher ORR compared with standard chemotherapy in the TROPiCS-02 and TROPION-Breast01 trials, respectively [28,30]. Despite targeting the same antigen—TROP2—the two agents differ substantially in toxicity profile and dosing schedule, and these distinctions should inform treatment selection [28,30].

While some may argue that the absence of an OS benefit in TROPION-Breast01 favors the use of SG, interpretation of these findings requires caution. It is important to note the differences in the populations enrolled in these trials: TROPION-Breast01 evaluated a less heavily pretreated cohort (1–2 prior chemotherapy lines), while TROPiCS-02 included patients with more extensive prior treatment (2–4 chemotherapy lines). A higher proportion of patients in the chemotherapy control arm of TROPION-Breast01 received subsequent ADCs (24% vs. 12% in the Dato-DXd arm), which may have confounded OS outcomes [28,30].

A real-world analysis presented at SABCS 2024 further explored the comparative performance of SG and Dato-DXd in mBC. Although the analysis did not stratify results by subtype (TNBC vs. HR-positive, HER2-negative), it included pooled data from nine clinical trials—three for Dato-DXd and six for SG—totaling 1281 patients. No statistically significant differences were observed in PFS (HR 0.95) or OS (HR 1.06) between the two agents. However, SG was associated with higher rates of grade ≥3 hematologic toxicities, including neutropenia (49% vs. 1%) and anemia (19% vs. 1.7%), while Dato-DXd was more frequently associated with stomatitis (60.1% vs. 11.9%) and ILD (2.6%). These findings reinforce the importance of individualizing ADC selection based on patient-specific comorbidities and toxicity risk profiles [69].

Looking forward, the DESTINY-Breast15 study (NCT05950945) aims to recruit patients with HER2-null disease, including those without observable membrane staining, potentially broadening T-DXd application to any level of HER2 expression. Additionally, patritumab deruxtecan, a HER3-directed ADC whose activity is independent of HER2 expression, is being evaluated in populations that include HER2-null tumors (NCT07060807).

### 6.2 TNBC

In pretreated metastatic TNBC, SG, T-DXd, and sac-TMT (approved only in China) have all demonstrated superiority over standard chemotherapy in phase 3 trials. However, the strength of evidence differs: SG is the only ADC validated in a randomized phase 3 trial enrolling a broad, geographically diverse population, whereas sac-TMT was evaluated exclusively in East Asian centers and T-DXd's TNBC activity derives from an exploratory subgroup analysis of HER2-low patients in DESTINY-Breast04. Accordingly, the ESMO expert consensus recommended prioritizing SG over T-DXd for HER2-low TNBC, with over 90% panelist agreement [70].

For PD-L1–positive TNBC, SG plus pembrolizumab represents a new first-line strategy.51 For patients ineligible for immunotherapy (PD-L1–negative or clinical contraindications), both SG and Dato-DXd emerge as treatment options. Cross-trial comparisons between TROPION-Breast02 (Dato-DXd) and ASCENT-03 (SG) are limited by population differences, shorter follow-up for SG, and permitted crossover in ASCENT-03 that may confound OS analysis. Nonetheless, available data highlight distinct profiles: Dato-DXd showed a higher ORR (62.5% vs. 48%), an OS benefit, and lower grade ≥3 adverse events (33% vs. 66%), while SG requires two infusions per cycle (days 1 and 8) compared with one for Dato-DXd. In practice, treatment selection will depend on patient preferences, comorbidity-driven toxicity considerations, and infusion logistics [29,33]. Other ongoing phase III trials may further redefine the first-line treatment landscape for TNBC. (Table 3B).

## 7 Special considerations

### 7.1 Importance of pathology

A critical aspect of T-DXd treatment selection is accurate HER2 pathology assessment, as HER2 status is frequently misclassified. In a biopsy reanalysis, 65.8% of patients initially scored as HER2-null were reclassified as HER2-low (IHC 1+) or ultralow, expanding the pool of T-DXd candidates [71]. Similarly, in DESTINY-Breast06, 64% of patients with local HER2-null scores were centrally reclassified as HER2-low (24%) or HER2-ultralow (40%) [72]. These findings highlight the need for more quantitative and standardized methods to detect low-level HER2 expression and to establish the minimum HER2 threshold (if any) for T-DXd efficacy. Given tumor heterogeneity, repeat biopsies should also be considered, as the likelihood of identifying HER2-low status increases with additional sampling [73].

These reclassification rates reflect well-documented interobserver variability observed mainly between IHC 0 and 1+, where multicenter studies report full agreement in fewer than 5% of cases [74,75]. Pre-analytic factors—including prolonged cold ischemia, fixation variability, and biopsy-to-excision discordance further compromise low-level HER2 detection. The 2023 ASCO/CAP guideline update acknowledged that current IHC assays were optimized to detect overexpression and that the 0 and 1+ threshold is an artifactual boundary rather than a validated predictive cutoff [76]. AI-assisted scoring has shown promise, improving interobserver agreement at the 0 and 1+ boundary from 69.8% to 87.4% in multicenter studies, though significant variability across independently developed AI models (median pairwise agreement 65.1%) highlights the need for standardized validation before clinical adoption [77,78].

### 7.2 Sequencing after an ADC

Optimal ADC sequencing remains an unresolved challenge. Resistance mechanisms are multifactorial, including reduced target antigen expression, impaired intracellular trafficking and lysosomal processing, alterations in payload targets, and upregulation of drug efflux transporters (ABCB1, ABCG2) [[79], [80], [81], [82], [83]]. Cross-resistance appears more strongly associated with shared payload mechanisms than target antigen loss, supporting payload class switching when possible; however, the degree of cross-resistance between topoisomerase I inhibitor-based ADCs remains incompletely characterized [82].

In addition to sequential ADC strategies, alternative treatment approaches remain relevant after ADC progression. In HR-positive, HER2-negative disease, endocrine-based therapy rechallenge may be considered in selected patients with favorable disease biology and may offer a chemotherapy-free interval. Chemotherapy also remains an important treatment option across all breast cancer subtypes and can be considered based on disease burden, prior therapies, and patient preferences.

Real-world data report median PFS of approximately 2–3 months with a second ADC, with declining efficacy when sequential agents share the same payload class and intervening chemotherapy has not consistently improved outcomes [[84], [85], [86]]. However, these data are derived from retrospective analyses with inherent limitations, including selection bias, heterogeneous prior treatment lines, and small sample sizes, and should be interpreted with caution. Prospective studies such as the phase II TRADE-DXd trial (NCT06533826), evaluating T-DXd and Dato-DXd sequencing, may help define future approaches and identify novel biomarkers.

### 7.3 Biomarkers of response and resistance

The identification of reliable predictive biomarkers remains an unmet need. For T-DXd, HER2 expression is the most established determinant of response; the phase II DAISY trial demonstrated a gradient of efficacy across HER2-overexpressing, HER2-low, and HER2 non-expressing subgroups [87]. Emerging data suggest ERBB2 amplification by comprehensive genomic profiling and quantitative HER2 assays may further refine patient selection [[87], [88], [89], [90]]. Genomic alterations including PTEN mutations and CDK12 deletions have been associated with inferior outcomes, while HER2 IHC discordance between sequential biopsies may reflect dynamic expression changes contributing to resistance [88,89]. For SG, TROP2 expression has not been validated as a predictive biomarker; benefit was observed across TROP2 subgroups in ASCENT and TROPiCS-02, though numerically greater efficacy was seen with higher expression [30,31]. TROP2 testing is therefore not currently required before SG administration. Emerging approaches such as multiplex immunofluorescence assays quantifying HER2 and TROP2 simultaneously, composite models like the ADC Treatment Response Score (ADC-TRS), and prospective studies evaluating dynamic biomarkers (ctDNA, PAM50, CelTIL) may further support ADC selection and sequencing strategies [91,92].

### 7.4 Patients with central nervous system metastasis (CNS)

CNS metastases in HER2-negative mBC remain a significant challenge due to limited ADC efficacy data, with treatment strategies often extrapolated from HER2-positive disease. The DESTINY-Breast12 trial demonstrated encouraging CNS activity with T-DXd in HER2-positive disease (12-month CNS PFS 58.9%; intracranial ORR 71.7%), but HER2-low patients were excluded [93].

Emerging data from the phase II DEBBRAH study (NCT04420598) have begun to address this gap. In HER2-low patients with active brain metastases (n = 12), T-DXd achieved intracranial ORRs of 50.0% and 33.3% across cohorts, with a median PFS of 5.4 months [94]. In patients with leptomeningeal carcinomatosis (n = 7; 3 HER2-positive, 4 HER2-low), median PFS was 8.9 months, with 71.4% achieving prolonged stabilization (≥24 weeks) [95]. By contrast, real-world data evaluating SG in 33 patients with brain metastases from HER2-negative breast cancer showed more modest intracranial activity (CNS ORR 13%; median CNS-PFS 2.9 months) [96].

Beyond approved ADCs, the TUXEDO-3 trial (NCT05865990) demonstrated promising CNS activity of patritumab deruxtecan (HER3-DXd) across breast cancer subtypes, with an intracranial ORR of 23.8% in brain metastases (n = 21) and intracranial clinical benefit rate of 50.0% in leptomeningeal disease (n = 20) [97,98]. These findings highlight the need for prospective, dedicated trials evaluating CNS activity of ADCs in HER2-negative mBC. Ongoing studies including DATO-Base (NCT06176261), TUXEDO-2 (NCT05866432), and BERLIN (NCT07458113) are further evaluating CNS-active ADC strategies. Until such data are available, local therapies remain the standard of care, with treatment decisions individualized based on extent of CNS involvement, prior treatments, and patient factors.

## 8 Future perspectives

The ADC landscape in breast cancer is expanding beyond HER2 and TROP2, with novel targets including HER3, Nectin-4, LIV-1, B7-H4, and folate receptor-α under active investigation. Among these, patritumab deruxtecan (HER3-DXd) has demonstrated promising early-phase activity [99]. Beyond novel targets, next-generation ADC platforms are also emerging, including bispecific ADCs—such as the EGFR×HER3-directed izalontamab brengitecan—as well as immune-stimulating conjugates, molecular glue–antibody conjugates, and multi-payload ADCs [[100], [101], [102]]. ADC-immunotherapy combinations are also advancing, as demonstrated by the ASCENT-04/KEYNOTE-D19 trial [34,99]. Together with advances in linker technology, site-specific conjugation, and dynamic biomarkers such as ctDNA and AI-driven analyses, these strategies may further optimize ADC selection, overcome resistance, and redefine the treatment landscape [102].

## 9 Conclusion

The advent of ADCs has introduced a groundbreaking therapeutic strategy for HER2-negative mBC. T-DXd, SG, sac-TMT, and Dato-DXd have demonstrated clinically meaningful improvements over standard chemotherapy, positioning ADCs as relevant therapeutic options for this population. ADC-based therapy is progressively redefining the treatment paradigm, not only as alternatives to conventional chemotherapy but also as the foundation for novel combination regimens and refined sequencing strategies. Future clinical trials will be essential to clarify optimal ADC positioning and to integrate these agents into personalized therapeutic approaches.

## Clinical trial number

Not applicable.

## Funding sources

None.

## CRediT authorship contribution statement

**Mariana Carvalho Gouveia:** Conceptualization, Writing – original draft. **Pedro José Galvão Freire:** Writing – original draft. **Caio Guilherme Souza Prado:** Writing – original draft. **Caio Abner Leite:** Writing – original draft. **Bruna Migliavacca Zucchetti:** Writing – review & editing. **Renata Colombo Bonadio:** Writing – review & editing. **Carlos Barrios:** Writing – review & editing. **Adriana Kahn:** Writing – review & editing. **Maryam Lustberg:** Writing – review & editing. **Romualdo Barroso-Sousa:** Conceptualization, Writing – review & editing.

## Declaration of competing interests

**MCG-** Speaker fees: Novartis, Knight Therapeutics, GSK, AstraZeneca, Abbvie, Daiichi Sankyo; Financial support for educational programs and symposia: Pfizer, Abbvie, GSK, AstraZeneca.

**PJGC-** Speaker fees- Novartis, Eli Lilly, Gilead, MSD/Merck, Servier, Dr. Reddy's; Financial support for educational programs and symposia: Novartis, AstraZeneca, Daiichi-Sankyo, Eli Lilly, Gilead, MSD/Merck, Servier, Hoffman La Roche; Research funding (to the institution): AstraZeneca, Hoffman La Roche, MSD/Merck, Novartis, GSK.

**CGSP-** No conflicts to declare.

**CAL-** Speaker fees- Bayer and AstraZeneca.

**BMZ-** Speaker fees- MSD, Novartis, Lilly-Eli, Astrazeneca, Zodiac, Pfizer, Daiichi-Sankyo; Advisory Board: Astrazeneca, Novartis, Lilly-Eli, Gilead; Research funding (to the institution): Astrazeneca.

**RCB-** Speaker fees and/or honoraria for consulting or advisory functions: Daiichi-Sankyo, Nestle Health Science, Addium, Gilead, MSD, BMS, AstraZeneca, Ache, Pfizer, Roche, Novartis, Libbs, Lilly. Financial support for educational programs and symposia: AstraZeneca, Daiichi-Sankyo, MSD, Lilly. Institutional Research grant: Novartis, AstraZeneca.

**CB-** CHB: Grants/research support: (to the institution) Amgen, Astra Zeneca, Aveo Oncology, BioNTech, BMS, Daiichi Sankyo, Dizal Pharma, Exelixis, Fortrea, Gilead Sciences, GSK, ICON, IQVIA, Janssen, Labcorp, Lilly, Medpace, MSD, Novartis, Novocure, Nuvisan, OBI Pharma, Parexel, Pfizer, PharmaMar, PPD, PSI, Regeneron, Roche/Genentech, Samsung, Sandoz, Sanofi, Seagen, Servier, Stemline, Syneos Health, Taiho, Takeda, Tolmar, TRIO, Worldwide. Academic Research Projects: CPO, PUCRS, LACOG, GBECAM. Ownership or Stocks: Tummi, MEDSir, CPO. Advisory Boards, Consulting, Travel, Presentation Honoraria: Adium, Novartis, Pfizer, Roche/Genentech, MSD, Astra Zeneca, Lilly, Daiichi, Gilead. SLG: Stock Ownership (Current, Self) HCA Healthcare; Consulting (All Relationships Ended, Self): Pfizer, SeaGen, AstraZeneca, DaiichiSankyo, Gilead Sciences, Menarini Stemline, Genentech, Novartis, Lilly/Loxo@Lilly; Research Funding (All Funds to Institution): AstraZeneca, Novartis, Daiichi Sankyo, Lilly, Hoffmann-La Roche/Genentech.

**AK-** Principal investigator on funded clinical trials grants: BMS, AstraZeneca, MSD/Merck.

**ML-** Consulting activities: AstraZeneca, Daiichi-Sankyo, Novartis, Lilly, Gilead and Menarini.

**RBS-** Speaker fees and/or honoraria for consulting or advisory functions: AstraZeneca, Daiichi-Sankyo, Eli Lilly, Gilead, Libbs, Pfizer, Novartis, MSD, and Roche. Financial support for educational programs and symposia: AstraZeneca, Daiichi-Sankyo, Gilead, Eli Lilly, and MSD. Institutional research grant: AstraZeneca, Daiichi-Sankyo.
